# Which factors are helpful for the early determination of treatment level in patients with interstitial lung disease in the intensive care unit to minimize the suffering in their end of life?: A retrospective study

**DOI:** 10.1097/MD.0000000000030524

**Published:** 2022-09-16

**Authors:** Sun-Hyung Kim, Dong-Hwa Lee, Bumhee Yang, Jun Yeun Cho, Hyeran Kang, Kang Hyeon Choe, Ki Man Lee, Yoon Mi Shin

**Affiliations:** a Department of Internal Medicine, Chungbuk National University Hospital, Chungbuk National University College of Medicine, Cheongju, South Korea.

**Keywords:** intensive care unit, interstitial lung disease, mechanical ventilation, prognosis

## Abstract

Interstitial lung disease (ILD) is widely known to be associated with high mortality and poor prognosis, especially in patients admitted to the intensive care unit (ICU). The objective of this study was to investigate clinical predictors for assisting relatively early decision of treatment level in the ICU. We retrospectively investigated patients with ILD who were admitted to the ICU between January 1, 2014, and September 30, 2019. A total of 64 patients were analyzed. We found the ICU and hospital mortality rates to be 67.2% and 69.8%, respectively. Nonsurvivors had a higher fraction of inspired oxygen (FiO_2_) on days 1 (79 ± 21 vs 60% ± 21%, *P* = .001) and 3 (61 ± 31 vs 46% ± 19%, *P* = .004). They showed lower partial pressure of oxygen/FiO_2_ (PF) ratio on days 1 (134 ± 80 vs 173 ± 102, *P* = .049) and 3 (147 ± 74 vs 235 ± 124, *P* = .003) than the survivor group. The lactic acid levels obtained on day 1 and PF ratio measured on day 3 were associated with mortality (odds ratio, 1.89; 95% confidence interval 1.03–3.47 and odds ratio, 0.99; 95% confidence interval 0.98–1.00, respectively). Among the 31 ICU survivors, 10 patients died in the general ward, 12 patients died after hospital discharge; only 9 patients survived after 1 year. We suggest that these clinical predictors could be used to determine the level of further treatment or withdrawal on day 3 of admission in patients with ILD admitted to the ICU to minimize the prolonged suffering in a relatively early period.

## 1. Introduction

The mortality in interstitial lung disease (ILD) is between 41% to 65.9% in the event of acute exacerbation that requires admission to the intensive care unit (ICU).^[1–3]^ According to a recent meta-analysis, the mortality of patients with ILD, including idiopathic pulmonary fibrosis (IPF), admitted to the ICU was approximately 50%.^[4]^ However, the mortality is higher in several patients with ILD admitted to the ICU. Currently, palliative care is implemented in patients with chronic lung diseases, such as chronic obstructive pulmonary disease, ILD, and all types of cancer.^[5]^

A previous study found that most patients with IPF (93%) were hospitalized during the last 6 months of their lives, and the hospital was the place of death for 80% of the enrolled patients.^[6]^ Patients having end of life discussions are willing to discontinue life-sustaining treatment. They are likely to refuse entry into the ICU and instead prepare for end of life with their family.^[7]^ However, patients agree to admission to the ICU if their own or their family’s awareness of the natural history of the disease is insufficient or if the patient visits the emergency room due to acute exacerbation. Intensive care is inevitably accompanied by different types of pain and distress to the patient and exerts an economic burden on their family.^[8]^ The economic burden imposed on patients with IPF continues to increase, especially in those admitted to the ICU and receiving mechanical ventilation (MV).^[9]^ Intensivists must always prioritize patients who require ICU admission in addition to the requisite arrangements, owing to the limitation in the number of beds and various equipment. However, these patients usually require longer ICU admission, even if their clinical course shows poor results. Furthermore, intensive care is often discontinued unilaterally by the family while the patient is undergoing MV or if the patient’s condition has deteriorated before their eventual death.

The law on the withdrawal of life-sustaining treatment was enacted in 2018 in South Korea, which makes provisions for the discontinuation of life-sustaining treatment in patients in the end-of-life process, that is, those who are not likely to recover. According to a meta-analysis on prognosis and risk factors of ILD patients admitted in the ICU, MV support was associated with mortality regardless of ILD etiology. Hypoxemia and APACHE score were associated with increased mortality in all ILDs, except for IPF.^[4]^ However, despite these previous results, there are no proven guidelines to assess the prognosis of patients with ILD admitted in the ICU. Moreover, there is no index that provides prognosis in early period of ICU admission to decide further treatment level in these high mortality patients. Therefore, it is necessary to analyze the clinical data of these patients to determine the prognostic factors and hope that it serves as a good objective basis for deciding whether to perform or discontinue intensive care. The purpose of this study was to evaluate the prognosis and investigate clinical predictors for assisting relatively early decision of treatment in patients with ILD in the ICU.

## 2. Methods

### 2.1. Study population

This retrospective study included all patients with ILD who were treated at the ICU of Chungbuk National University Hospital between January 1, 2014, and September 30, 2019. Patients admitted to the ICU for short-term intensive observation after surgery or those who were hospitalized for other diseases were excluded. A total of 64 patients were analyzed.

This study was approved by the Institutional Review Board of Chungbuk National University Hospital, South Korea (IRB No. 2020-02-012) and was conducted in accordance with the amended Declaration of Helsinki. The need for informed consent was waived because no patients were placed at risk during this study.

### 2.2. Data collection

All medical information and laboratory data were collected from the medical records of each patient as follows: demographic characteristics, comorbidities before ICU admission, chest computed tomography findings, findings of previous pulmonary function tests (PFTs), treatment modalities, ICU mortality, lengths of ICU and hospital stay, and ventilator settings. The fraction of inspired oxygen (FiO_2_) and ratio of the partial pressure of oxygen to FiO_2_ (partial pressure of oxygen/FiO_2_ [PF]) were measured on days 1 and 3 of the ICU stay. Positive end-expiratory pressure and driving pressures were measured on days 1 and 3 in patients undergoing MV. We analyzed and compared the data of the survivors and nonsurvivors. We also analyzed the subgroup data of patients with IPF.

The ILD subtype was classified according to the American Thoracic Society/European Respiratory Society International Multidisciplinary Classification of Idiopathic Interstitial Pneumonia.^[10]^ The definition of acute exacerbation of ILD was based on the acute exacerbation of IPF provided by the International Working Group Report.^[11]^ Acute exacerbation of IPF is an acute, clinically significant respiratory deterioration characterized by evidence of new widespread alveolar abnormality. Diagnostic criteria are as follows; Acute worsening or development of dyspnea should occur within 1 month. A new bilateral ground glass opacity (GGO) or consolidation should be superimposed on the existing ILD pattern on chest tomography. The deterioration cannot be explained by cardiac failure and fluid overloading.

Organ failure was measured using the Sequential Organ Failure Assessment (SOFA) score,^[12]^ and the severity of acute respiratory distress syndrome was classified according to the Berlin criteria.^[13]^

### 2.3. Statistical analysis

Continuous variables were expressed as the mean and standard deviation or as median and interquartile range, while categorical variables were expressed as numbers (%). The chi-squared test was used for the comparison of the categorical variables. The Mann–Whitney *U* test was used for inter-group comparison of the continuous variables. We analyzed the age, sex, comorbidities, ILD subgroups, PFTs, ICU Glasgow Coma Scale score (GCS) score, SOFA score, initial laboratory findings, and treatment modalities using univariate analysis, and parameters with *P* values <.05 were further subjected to multiple logistic regression to evaluate their association with ICU mortality. Two-tailed *P* values <.05 were considered to indicate statistical significance. All statistical analyses were performed using IBM SPSS version 21.0 (IBM Corp., Armonk, NY).

## 3. Results

The baseline characteristics of the 64 patients with ILD at ICU admission are shown in Table 1. The median age was 72 years, and more than half of the patients were men (60.9%). Cardiovascular disease (54.7%) was the most common comorbidity, followed by diabetes mellitus. IPF (65.6%) was the most common subtype of ILD in this cohort, followed by autoimmune ILD. The PFT conducted within the last 1 year was used for analysis. We could obtain the PFT results of only 30 of the 64 patients. The average forced vital capacity was 2.17 L, the forced expiratory volume in 1 second was 1.81 L, and the forced expiratory volume in 1 second/forced vital capacity was 81%. There was no significant difference between the PFT results of the survivor and nonsurvivor groups. The diffusing lung capacity results were available in only 19 patients. Acute exacerbation (85.9%) of ILD was the most common cause of ICU admission. The initial median GCS and SOFA scores at ICU admission were 12 and 6, respectively. The GCS score was significantly higher in the survivor group (14 vs 11, *P* = .015), while the SOFA score was lower in the survivor group (4 vs 7, *P* = .021) compared with the nonsurvivor group. The severe type (45.3%) was the most common category of acute respiratory distress syndrome according to the Berlin criteria. The initial lactic acid level showed a significant difference in the survivor group (1.7 vs 2.3 mg/dL, *P* = .034). Subgroup analysis was also performed in patients with IPF (Table S1, Supplemental Digital Content 1, http://links.lww.com/MD/H281). The nonsurvivor group had higher SOFA scores and serum creatinine levels than the survivor group.

**Table 1:** Baseline characteristics of patients with interstitial lung disease on ICU admission.

Characteristics	Total (N = 64)	Survivors (N = 31)	Nonsurvivors (N = 33)	*P* value
Age	72 (67–77)	68 (53–83)	72 (67–79)	.554
Male	39 (60.9)	17 (54.8)	22 (66.7)	.332
BMI	22.5 (19.8–25.0)	22.2 (15.6–28.8)	22.6 (19.6–24.9)	.877
Poor performance status*	22 (34.4)	14 (45.2)	8 (24.2)	.078
Comorbidity				
Cardiovascular disease	35 (54.7)	16 (51.6)	19 (57.6)	.632
Diabetes mellitus	13 (20.3)	4 (12.9)	9 (27.3)	.217
Connective tissue disease	12 (18.8)	5 (16.1)	7 (21.2)	.603
Malignancy	12 (18.8)	7 (22.6)	5 (15.2)	.447
Neurologic disease	8 (12.5)	4 (12.9)	4 (12.1)	1.000
COPD	4 (6.3)	2 (6.5)	2 (6.1)	1.000
Chronic renal disease	3 (4.7)	1 (3.2)	2 (6.1)	1.000
ILD subgroups				
Idiopathic interstitial pneumonias				
IPF	41 (65.6)	21 (67.7)	20 (60.6)	.552
NSIP	8 (12.5)	3 (9.7)	5 (15.2)	.709
COP	4 (6.3)	1 (3.2)	3 (9.1)	.614
Autoimmune ILDs†	10 (15.6)	5 (16.1)	5 (15.2)	.914
Pulmonary function test within 1 year of admission	30/64			
FVC, L	2.17 (1.69–3.01)	1.86 (1.36–2.90)	2.43 (1.99–3.06)	.110
FVC, %	68.5 (51.8–82.0)	67 (49–81)	71 (57–83)	.473
FEV1, L	1.81 (1.37–2.43)	1.75 (1.22–2.41)	1.88 (1.69–2.46)	.355
FEV1, %	77 (61–88)	76 (53–87)	77 (65–89)	.552
FEV1/FVC, %	81 (75–84)	81 (73–85)	79 (77–85)	.951
Reason for ICU admission				.729
Acute exacerbation	55 (85.9)	26 (83.9)	29 (87.9)	
Extrapulmonary cause	9 (14.1)	5 (16.1)	4 (4.6)	
Heart failure	4 (6.2)	3 (4.6)	1 (1.6)	
Category for ARDS‡				.064
Mild	11 (17.2)	7 (22.6)	4 (12.1)	
Moderate	24 (37.5)	14 (45.2)	10 (30.3)	
Severe	29 (45.3)	10 (32.3)	19 (57.6)	
Initial status of ICU admission				
GCS score	13 (9–15)	14 (9–15)	11 (8–14)	.015
SOFA score	6 (4–9)	4 (3–8)	7 (5–9)	.021
Laboratory findings				
Serum creatinine, mg/dL	0.76 (0.48–0.96)	0.71 (0.45–0.86)	1.02 (0–2.30)	.056
hs-CRP, mg/dL	11.14 (4.08–20.40)	10.86 (4.09–17.65)	11.20 (3.10–26.16)	.973
Procalcitonin, ng/mL	0.17 (0–0.9)	0.24 (0–2.12)	0.16 (0–0.81)	.521
NT-proBNP, pg/mL	1546 (431–4928)	2775 (672–7398)	1441 (330–3073)	.39
Lactic acid, mg/dL	1.8 (1.2–2.6)	1.7 (1.2–2.3)	2.3(1.5–3.7)	.017

Data are presented as n (%) or the median (interquartile range).

ARDS = acute respiratory distress syndrome, BMI = body mass index, COP = cryptogenic organizing pneumonia, COPD = chronic obstructive pulmonary disease, CT = computed tomography, FEV1 = forced expiratory volume in 1 s, FVC = forced vital capacity, GCS score = Glasgow Coma Scale score, hs-CRP = high-sensitivity C-reactive protein, ICU = intensive care unit, ILD = interstitial lung disease, IPF = idiopathic pulmonary fibrosis, NSIP = nonspecific interstitial pneumonia, NT-proBNP = N-terminal prohormone B-type natriuretic peptide, SOFA score = sequential organ failure assessment score, .

* ECOG (Eastern Cooperative Oncology group) performance status class ≥3 means severe systemic disease with functional limitation.

† Autoimmune ILD included rheumatoid arthritis-associated interstitial lung disease (5 patients), systemic lupus erythematosus-associated interstitial lung disease (1 patient), Sjogren syndrome-associated interstitial lung disease (1 patient), systemic sclerosis-associated interstitial lung disease (1 patient), polymyositis-associated interstitial lung disease (1 patient), and antisynthetase syndrome-associated interstitial lung disease (1 patient).

‡ ARDS was defined using the Berlin criteria.

Table 2 shows the treatment modalities, clinical course, and outcomes in the ICU. All patients received antibiotics, and 51 patients (79.7%) received steroid treatment. Twenty-nine patients (45.3%) were administered vasopressor agents. The use of vasopressor agents was significantly higher in the nonsurvivor group than in the survivor group (32.3% vs 57.6% *P* = .042). In the clinical course, 10 patients (15.6%) developed pneumothorax. The incidence of pneumothorax was significantly higher in the nonsurvivor group than in the survivor group (3.2% vs 27.3% *P* = .013). Compared with the survivors, lactic acid was significantly higher in the nonsurvivors group on day 3 of ICU treatment (1.7 vs 2.0 mg/dL, *P* = .025).

**Table 2:** Treatment modality, clinical course, and outcomes.

	Total (N = 64)	Survivors (N = 31)	Nonsurvivors (N = 33)	*P* value
Treatments				
Antibiotics	64 (100)	31 (100)	33 (100)	
Steroids	51 (79.7)	25 (80.6)	26 (78.8)	.854
Use of vasopressor	29 (45.3)	10 (32.3)	19 (57.6)	.042
ECMO	5 (7.8)	0	5 (15.2)	.053
High flow nasal cannula	40 (62.5)	22 (71.0)	18 (54.5)	.175
Ventilator support	51 (79.7)	22 (71.0)	29 (87.9)	.124
CRRT	6 (9.4)	1 (3.2)	5 (15.2)	.198
Clinical course				
Presence of acute kidney injury	18 (28.1)	7 (22.6)	11 (33.3)	.339
Pneumothroax	10 (15.6)	1 (3.2)	9 (27.3)	.013
Lactic acid, mg/dL day 3	1.8 (1.2–2.1)	1.7 (1.2–2.0)	2.0 (1.4–2.9)	.025
Length of ICU stay, d	12 (4–17)	11 (4–19)	12 (6–17)	.877
Mortality		Survivors	Nonsurvivors	
ICU mortality	33/64 (51.6)	31	33	
In-hospital mortality	43/64 (67.2)	21	43	
30-day mortality after discharge	48/64 (75.0)	16	48	
1-year mortality after discharge	55/64 (85.9)	9	55	

Data are presented as n (%) or the median (interquartile range).

CRRT = continuous renal replacement therapy, ECMO = extracorporeal membrane oxygenation, ICU = intensive care unit.

A total 51 patients (79.7%) underwent MV. The median length of ICU stay was 12 days. The ICU mortality was 51.6%. Of the 33 patients who died in the ICU, a total of 28 (84.8%) agreed to treatment-limitation decisions just before their eventual death to avoid cardiac compression. In-hospital 30-day and 1-year mortality were 67.2%, 75%, and 85.9%, respectively. Subgroup analysis was performed in patients with IPF (Table S2, Supplemental Digital Content 2, http://links.lww.com/MD/H282).

**Figure 1.**
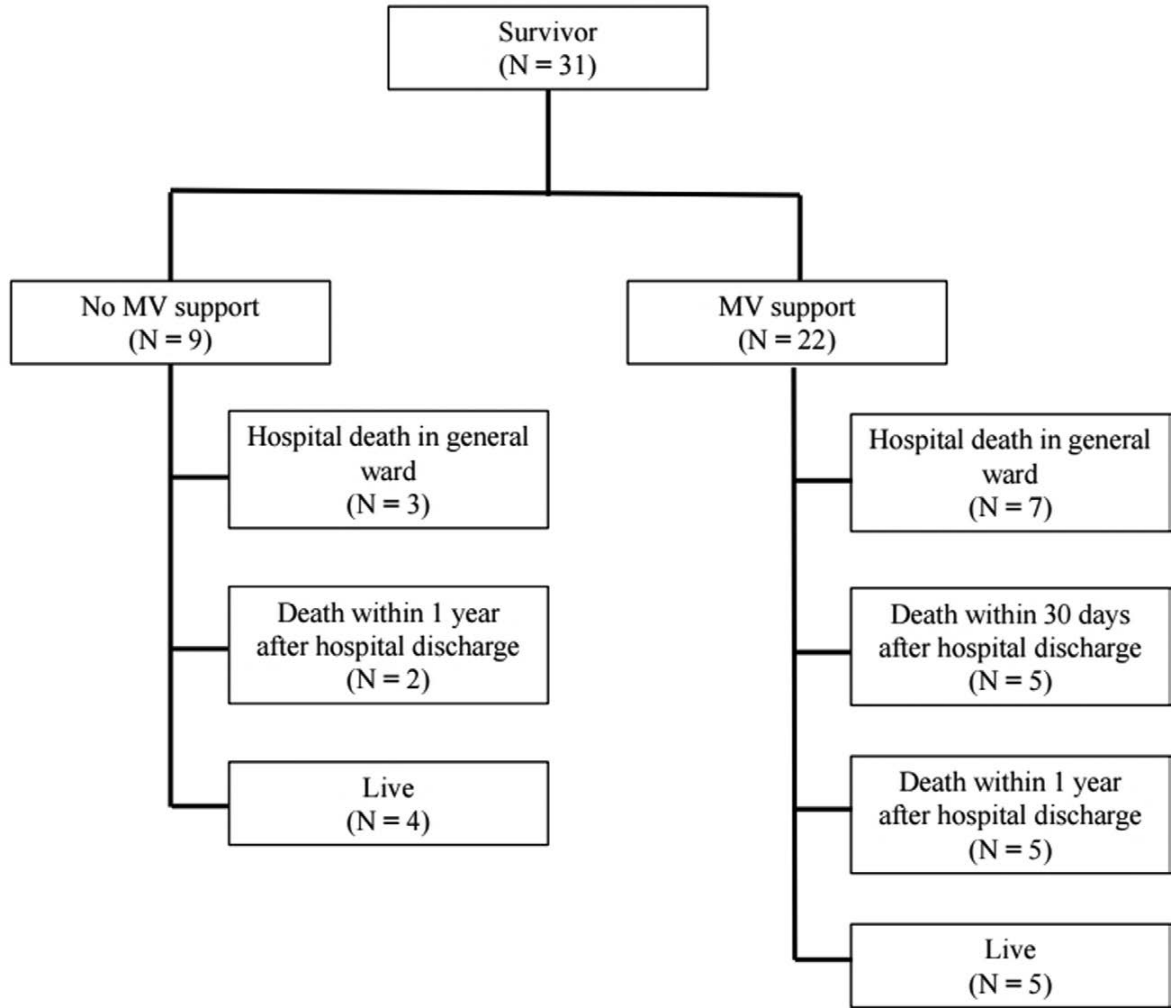
Long-term prognosis of ICU survivors. ICU = intensive care unit, MV = mechanical support.

**Figure 2.**
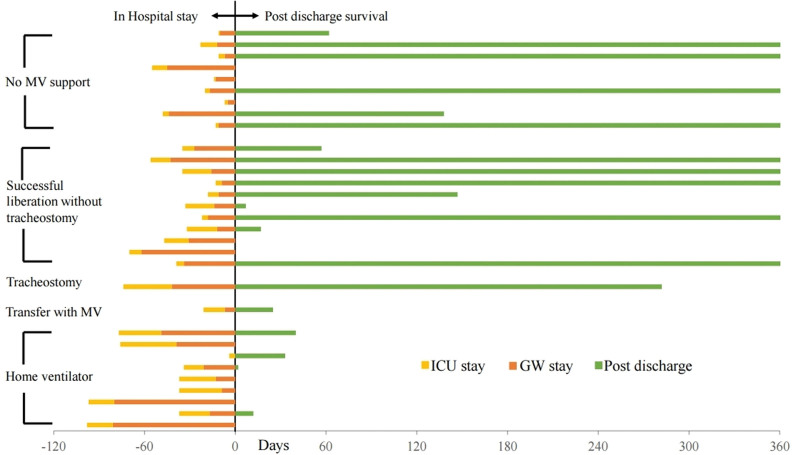
One-year survival bar graph in ICU survivors (N = 31) from the time of ICU admission. GW = general ward, ICU = intensive care unit, MV = mechanical support.

Figure 1 depicts the long-term prognosis of ICU survivors. For analyzing mortality, patients were divided into 2 groups: those who received MV and those who did not receive MV. Of the 31 ICU survivors, 10 patients died in the general ward. The hospital mortality was 67.2%. Additionally, 5 of these 10 patients died within 30 days of hospital discharge. Twelve patients died within 1 year of hospital discharge. The 1-year mortality rate of this study population was 85.9%. Figure 2 depicts the 1-year survival of ICU survivors from the time of ICU admission. Three patients who did not receive MV died in the hospital, 2 died within 1 year, and only 4 survived. Eleven of the 22 patients who received MV support were liberated without tracheostomy. Two patients died in the hospital, and 4 died within 1 year. Only 5 patients survived. Ten patients underwent tracheostomy, of whom 9 required a home ventilator. All patients who received home ventilator care died within 60 days of hospital discharge. One patient was transferred to other hospitals while maintaining MV.

**Figure 3.**
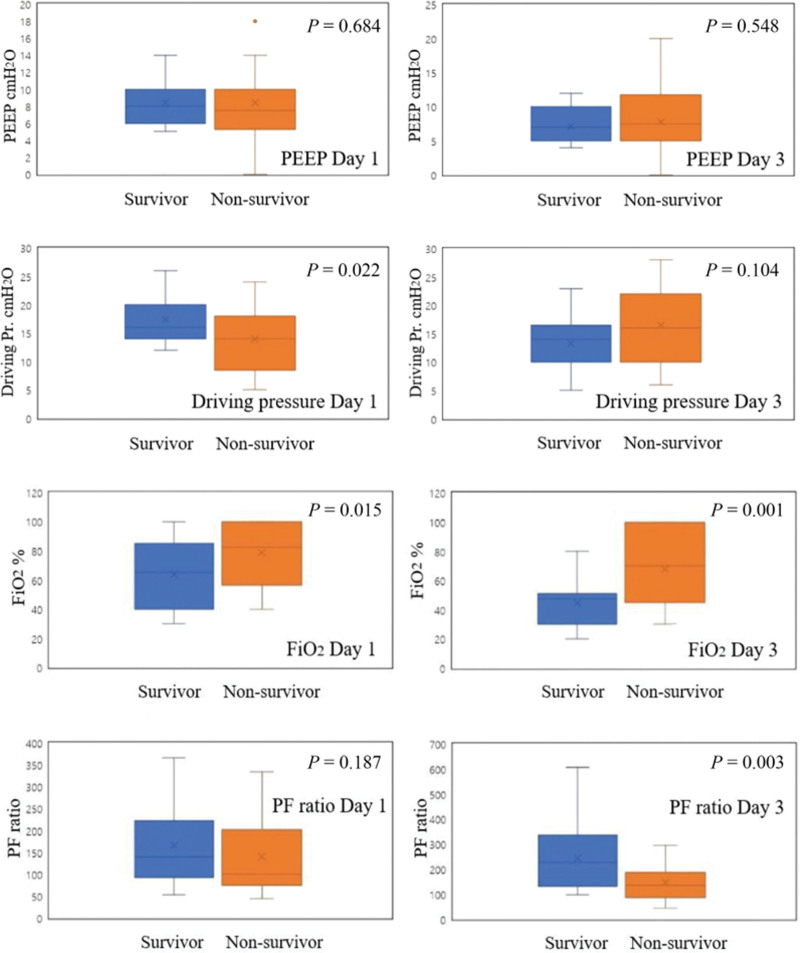
Comparison of ventilator settings between the survivor and nonsurvivor groups. FiO_2_ = fraction of inspired oxygen, PEEP = positive end-expiratory pressure, PF = ratio of the partial pressure of oxygen to FiO_2_, Pr. = pressure.

Table 3 depicts the comparison of oxygenation and ventilator settings between the survivor and nonsurvivor groups in the ICU. Nonsurvivors had a higher FiO_2_ on days 1 (83% vs 60%, *P* = .001) and 3 (60% vs 50% *P* = .004) in the ICU than the survivors. The PF ratio on day 1 (94 vs 155, *P* = .049) and day 3 (152 vs 225, *P* = .003) was significantly lower in the nonsurvivor group than in the survivor group. The ventilator settings were analyzed for 51 patients who underwent MV (Fig. 3). The driving pressure on day 1 was lower in the nonsurvivor group than in the survivor group (16 cmH_2_O vs 14 cmH_2_O, *P* = .022). The FiO_2_ on day 1 (65% vs 83%, *P* = .015) and 3 (48% vs 70%, *P* = .003) were significantly lower in the survivor group, and the PF ratio on day 3 (227 vs 153, *P* = .003) was higher in the survivor group than in the nonsurvivor group. No significant differences were observed in the other ventilator settings.

**Table 3:** Comparison of the oxygenation and ventilator settings between the survivors and nonsurvivors in the ICU.

All patients (N = 64)	Survivor (N = 31)	Nonsurvivor (N = 33)	*P* value
FiO_2_ %, Day 1	60 (40–80)	83 (65–100)	.001
PF ratio, Day 1	155 (94–222)	94 (74–195)	.049
FiO_2_ %, Day 3	50 (30–58)	60 (45–100)	.004
PF ratio, Day 3	225 (121–337)	152 (90–187)	.003
Only in patients with ventilator support (N = 51)	Survivor (N = 22)	Nonsurvivor (N = 29)	*P* value
Driving pressure cmH_2_O, Day 1	16 (14–20)	14 (9–18)	.022
PEEP cmH_2_O, Day 1	8 (6–10)	8 (5–10)	.834
Respiratory rate, Day 1	21 (17–25)	20 (17–24)	.934
FiO_2_%, Day 1	65 (40–85)	83 (56–100)	.015
PF ratio, Day 1	140 (93–222)	100 (74–203)	.187
Driving pressure cmH_2_O, Day 3	14 (10–17)	16 (10–22)	.104
PEEP cmH_2_O, Day 3	7 (5–10)	7 (5–11)	.548
Respiratory rate, Day 3	20 (18–24)	21 (20–28)	.835
FiO_2_%, Day 3	48 (30–51)	70 (45–100)	.001
PF ratio, Day 3	227 (130–335)	153 (89–189)	.003
Only in IPF patients (N = 41)	Survivor (N = 21)	Nonsurvivor (N = 20)	*P* value
FiO_2_%, Day 1	55 (40–78)	80 (55–100)	.020
FiO_2_ %, Day 3	50 (30–55)	75 (43–100)	.013
PF ratio, Day 3	227 (139–326)	109 (66–185)	.001

Data are presented as n (%) or the median (interquartile range).

FiO_2_ = fraction of inspired oxygen, PEEP = positive end-expiratory pressure, PF ratio = PaO_2_/FiO_2_ ratio.

Subgroup analysis was performed for patients with IPF alone (n = 41), and the results showed similar trends to those of patients with MV support. The FiO_2_ on days 1 and 3 and the PF ratio on day 3 differed significantly between the 2 groups.

Univariable analysis was performed on the factors that may affect ICU survival. By univariable analysis, the SOFA score, initial lactic acid level, lactic acid level on day 3, vasopressor support, occurrence of pneumothorax, FiO_2_ on day 1, FiO_2_ on day 3, and PF ratio on day 3 yielded a *P* value of <.05 for association with ICU mortality (Table 4). Only the PF ratio on day 3 (odds ratio, 0.99; 95% confidence interval 0.98–1.00) was independently associated with ICU mortality by multivariable analysis. The factors related to ICU mortality were also analyzed in patients who underwent MV. Univariable analysis revealed that the driving pressure on day 1, FiO_2_ on day 1, FiO_2_ on day 3, and PF ratio on day 3 yielded *P* values <.05 for the association with ICU mortality. Multivariable analysis revealed that only the FiO_2_ on day 3 was associated with ICU mortality (odds ratio, 1.05; 95% confidence interval 1.02–1.09).

**Table 4:** Results of logistic regression for the prognostic factors for ICU mortality.

	Univariate analysis		Multivariate analysis	
	OR (95% CI)	*P* value	OR (95% CI)	*P* value
Age	1.02 (0.96–1.08)	.544		
BMI	1.01 (0.89–1.16)	.868		
Male	0.61(0.22–1.67)	.334		
Poor performance status^a^	0.39 (0.13–1.13)	.082		
Cardiovascular disease	1.27 (0.48–3.41)	.632		
Diabetes Mellitus	2.53 (0.69–9.29)	.161		
Connective tissue disease	0.93 (0.21–4.10)	.925		
Malignancy	0.94 (0.12–7.08)	.949		
Neurologic disease	0.93 (0.21–4.10)	.925		
COPD	0.94 (0.12–7.08)	.949		
Chronic renal disease	1.94 (0.17–22.48)	.598		
IPF	0.73 (0.26–2.05)	.733		
NSIP	1.67 (0.36–7.65)	.511		
COP	3.00 (0.30–30.50)	.353		
Autoimmune ILDs	0.93 (0.24–3.58)	.914		
Criteria for Acute respiratory distress syndrome^c^				
Mild	1			
Moderate	1.25 (0.29–5.45)	.766		
Severe	3.33 (0.78–14.14)	.104		
ICU GCS	0.89 (0.79–1.01)	.077		
SOFA score	1.21 (1.02–1.44)	.033		
Serum creatinine, mg/dL	2.52 (0.90–7.08)	.079		
hs-CRP, mg/dL	1.01 (0.97–1.05)	.697		
Procalcitonin, ng/mL	1.02 (0.97–1.07)	.563		
NT-proBNP, pg/mL	1.00 (1.00–1.00)	.140		
Lactic acid, mg/dL, Day 1	1.54 (1.02–2.33)	.039		
Lactic acid, mg/dL, Day 3	2.06 (1.02–4.15)	.043		
Steroid	0.89 (0.26–3.02)	.854		
Vasopressor support	2.85 (1.03–7.92)	.045		
Acute kidney injury	1.71 (0.57–5.20)	.341		
CRRT	5.36 (0.59–48.73)	.136		
Pneumothorax	11.25 (1.33–95.10)	.026		
High flow nasal cannula	0.49 (0.17–1.38)	.178		
Ventilator support	2.94 (0.81–10.90)	.102		
FiO_2_ %, Day 1	1.04 (1.02–1.07)	.002		
PF ratio, Day 1	1.00 (0.99–1.00)	.107		
FiO_2_ %, Day 3	1.03 (1.00–1.06)	.014		
PF ratio, Day 3	0.99 (0.98–1.00)	.006	0.99 (0.98–1.00)	.006
In patients with MV				
Driving pressure cmH_2_O, Day 1	0.87 (0.77–0.99)	.028		
PEEP cmH_2_O, Day 1	1.00 (0.84–1.18)	.978		
Respiratory rate, Day 1	1.00 (0.90–1.11)	.952		
FiO_2_%, Day 1	1.03 (1.00–1.06)	.023		
PF ratio, Day 1	1.00 (0.99–1.00)	.283		
Driving pressurecmH_2_O, Day 3	1.10 (0.99–1.22)	.089		
PEEP cmH_2_O, Day 3	1.03 (0.89–1.20)	.681		
Respiratory rate, Day 3	1.09 (0.97–1.22)	.167		
FiO_2_%, Day 3	1.05 (1.02–1.09)	.003	1.05 (1.02–1.09)	.003
PF ratio, Day 3	0.99 (0.98–1.00)	.006		

95%CI = 95% confidence interval, BMI = body mass index, COP = cryptogenic organizing pneumonia, COPD = chronic obstructive pulmonary disease, CRRT = continuous renal replacement therapy, FiO_2_ = fraction of inspired oxygen, GCS score = Glasgow Coma Scale score, hs-CRP = high-sensitivity C-reactive protein, IPF = idiopathic pulmonary fibrosis, NSIP = nonspecific interstitial pneumonia, NT-proBNP = N-terminal prohormone B-type natriuretic peptide, OR = odds ratio, PEEP = positive end-expiratory pressure, PF ratio = PaO_2_/FiO_2_ ratio, SOFA score = sequential organ failure assessment score.

## 4. Discussion

The prognosis for patients with ILD who are admitted to the ICU is very poor. A systematic review reported that in-hospital mortality was observed in 52% of patients with mixed ILD and 68% of patients with IPF.^[4]^ Two studies conducted in South Korea reported that the hospital mortality of patients admitted to the ICU for acute exacerbation of IPF was 50% to 63%.^[14,15]^ In our study, the hospital mortality was 67.2%, which rose to 76% when patients with IPF were analyzed separately, higher than that of the aforementioned previous studies.

Most nonsurvivors and their family members agreed to treatment-limitation decisions just before their eventual death because they did not want cardiac compression. In South Korea, if the patient has no revivability according to doctor’s judgment, the patient and their family can choose the treatment modality, including transfusion, chemotherapy, ventilator care, vasopressor, extracorporeal membrane oxygenation, and cardiac compression, by the law for the withdrawal of life-sustaining treatment.

Previous studies reported that the 1-year mortality rate for patients with ILD admitted to the ICU ranged from 53% to 100%.^[4,16]^ The 1-year mortality rate in our study was 85.9%, similar to that of previous studies. One study reported that hospital mortality was higher in patients who underwent MV,^[16]^ although no significant difference was observed in this study. Similarly, this study demonstrated that the performance status was poor in survivors. Fifteen of the 28 patients who were liberated from the ICU MV, including the nonsurvivors who died after ICU discharge, underwent tracheostomy, and 9 required long-term maintenance on a home ventilator. These patients cannot return to their daily lives and spend the rest of their lives at the nursing hospital.^[17]^ They experience problems with respiration, nutrition, communication, and accidental decannulation and complain of fear of tube suction. This condition places a psychological and economic burden on the family and the patients.^[18,19]^

Several studies have endeavored to seek indicators related to the prognosis of patients with ILD admitted to the ICU. Some studies reported that ICU mortality increases with age, while others found no relationship between age and mortality.^[14,20]^ In our study, age did not affect prognosis.

In our study, the GCS and SOFA scores at ICU admission were significantly different between the survivor and nonsurvivor groups. The initial and day 3 lactic acid levels were quite different. The initial creatinine level was higher (*P* = .056) in the nonsurvivor group and was significantly higher in the IPF subgroup of nonsurvivors (*P* = .031). The use of vasopressor agents was substantially higher in the nonsurvivor group.

Previous studies have also shown that hypoxemia was related to mortality^[20,21]^; however, other studies have reported that the PF ratio was not a prognostic factor for 90-day survival.^[22]^ This study found that the survivor group had significantly better PF ratios and FiO_2_ on days 1 and 3 than the nonsurvivor group. According to the multivariate analysis, the PF ratio on day 3 was predictive of ICU mortality. A recent study reported that poor prognosis was associated with worsening oxygenation on days 5 and 7 after the onset of acute respiratory failure.^[3]^ Our study provided an early predictor to guide the nature of further treatment than that of previously reported data.

Pneumothorax occurred more frequently in the nonsurvivor group in this study. According to the univariate analysis, pneumothorax was associated with ICU mortality, although this significance was not sustained in the multivariate analysis. Studies have reported poor prognosis in patients with IPF and pneumothorax,^[22]^ and similar results were observed in this study.

This study had some limitations. First, this retrospective study was conducted at a single center. Second, our study incorporated a relatively small sample size, the majority of which comprised patients with IPF.

In conclusion, our study also observed that the ICU and in-hospital mortality was high in patients with ILD admitted to the ICU. In these patients, we found that the initial GCS and SOFA scores, lactic acid levels on days 1 and 3, use of vasopressors, and occurrence of pneumothorax were associated with poor prognosis, while the PF ratios measured on day 3 were associated with mortality.

Therefore, we suggest that these clinical predictors could be used to determine the level of further treatment or withdrawal on day 3 of admission in patients with ILD admitted to the ICU to minimize the prolonged suffering in a relatively early period.

## Acknowledgments

The authors wish to thank the staff of the medical ICU for their continuous support during the treatment of the patients.

## Author contributions

**Conceptualization:** Yoon Mi Shin.

**Data curation:** Sun-Hyung Kim, Bumhee Yang, Jun Yeun Cho, Hyeran Kang, Hyeran Kang, Kang Hyeon Choe, Ki Man Lee, Yoon Mi Shin.

**Formal analysis:** Sun-Hyung Kim, Dong-Hwa Lee, Yoon Mi Shin.

**Investigation:** Sun-Hyung Kim, Yoon Mi Shin.

**Methodology:** Yoon Mi Shin.

**Project administration:** Dong-Hwa Lee.

**Resources:** Yoon Mi Shin.

**Supervision:** Bumhee Yang, Jun Yeun Cho, Hyeran Kang, Kang Hyeon Choe, Ki Man Lee, Yoon Mi Shin.

**Validation:** Yoon Mi Shin.

**Writing – original draft:** Sun-Hyung Kim, Dong-Hwa Lee, Yoon Mi Shin.

**Writing – review & editing:** Sun-Hyung Kim, Dong-Hwa Lee, Yoon Mi Shin.

## Supplementary Material


