# Supplementary material for: Which factors are helpful for the early determination of treatment level in patients with interstitial lung disease in the intensive care unit to minimize the suffering in their end of life?: A retrospective study
Source: Medicine (Baltimore). 2022 Sep 16;101(37):e30524. doi: 10.1097/MD.0000000000030524 (PMC9478284; doi:10.1097/MD.0000000000030524)
Supplement: Supplementary file 1 [file medi-101-e30524-s001.pdf]

Supplemental Table 1. Baseline characteristics of patients with IPF at ICU admission and outcomes.

Total (N=41)	Median (IQR) or N (%)	Survivor (N=21)	Non-Survivor (N=20)	*P*-value
Demographics				
Age	73 (68-77)	73 (68-77)	73 (68-78)	0.990
Male	33 (78.6)	15 (71.4)	17 (85.0)	0.454
BMI	22.3 (19.7-24.6)	22.1 (20.0-24.4)	22.7 (19.5-24.9)	0.824
Poor performance status[a]	13 (31.7)	9 (42.9)	4 (20.0)	0.181
Comorbidity				
Cardiovascular disease	20 (48.8)	9 (42.9)	11 (55.0)	0.437
Diabetes mellitus	10 (24.4)	4 (19.0)	6 (30.0)	0.484
Malignancy	9 (22.0)	4 (19.0)	5 (25.0)	0.719
Neurologic disease	5 (12.2)	3 (14.3)	2 (10.0)	1.000
COPD	4 (9.8)	2 (9.5)	2 (10.0)	1.000
Connective tissue disease	1 (2.4)	0	1 (5.0)	0.488
Chronic renal disease	2 (4.9)	0	2 (10.0)	0.232
Reason for ICU admission				
Acute exacerbation	36 (87.8)	18 (85.7)	18 (90.0)	1.000
Extra-pharenchymal cause	5 (12.2)	3 (14.3)	2 (10.0)	1.000
CT findings at ICU admission				
Honeycombing	40 (97.6)	21 (100)	19 (95.0)	0.488
Ground-glass opacities	32 (78.0)	15 (71.4)	17 (85.0)	0.454
Air-space consolidation	29 (70.7)	16 (76.2)	13 (65.0)	0.505
Criteria for acute respiratory distress syndrome[b]				
Mild	6 (14.3)	4 (19.0)	2 (10.0)	0.279
Moderate	18 (42.9)	11 (52.4)	7 (35.0)	0.279
Severe	18 (42.9)	6 (28.6)	11 (55.0)	0.279
GCS score	12 (8-14)	14 (7-15)	11 (9-14)	0.080
SOFA score	7 (4-10)	5 (3-10)	8 (6-10)	0.046
Laboratory findings				
Serum creatinine, mg/dL	0.8 (0.6-1.0)	0.7 (0.4-0.9)	0.9 (0.7-1.5)	0.031
hs-CRP, mg/dL	11.0 (4.4-19.8)	9.9 (4.3-16.8)	12.6 (4.3-27.6)	0.348
Procalcitonin, ng/mL	0.14 (0-1.12)	0.14 (0-2.09)	0.14 (0-1.12)	0.853
NT-proBNP, pg/mL	2305 (1018-50503)	3244 (1108-7398)	1542 (652-3751)	0.326
Lactic acid, mg/dL	1.8 (1.3-2.7)	1.7 (1.2-2.2)	2.5 (1.5-3.7)	0.066

Pulmonary function test	24/41			
FVC, L	2.63 (1.73-3.13)	1.99 (1.38-3.04)	2.88 (2.12-3.17)	0.156
FVC, %	71 (59-82)	69 (51-88)	72 (67-82)	0.750
FEV1, L	1.97 (1.43-2.51)	1.89 (1.28-2.52)	2.21 (1.73-2.53)	0.487
FEV1, %	79 (67-89)	79 (60-91)	77 (72-87)	0.908
FEV1/FVC, %	80 (75-84)	80 (72-86)	78 (77-82)	0.931

COPD = chronic obstructive pulmonary disease, FVC = forced vital capacity, CT = computed tomography, FEV1 = forced expiratory volume in 1 s, GCS score = Glasgow Coma Scale score, SOFA score = Sequential Organ Failure Assessment score, hs-CRP = high-sensitivity C-reactive protein

[a]ECOG (Eastern Cooperative Oncology group) performance status class ≥3 means severe systemic disease with functional limitation.

[b]ARDS was defined using the Berlin criteria.
