# Supplementary material for: Which factors are helpful for the early determination of treatment level in patients with interstitial lung disease in the intensive care unit to minimize the suffering in their end of life?: A retrospective study
Source: Medicine (Baltimore). 2022 Sep 16;101(37):e30524. doi: 10.1097/MD.0000000000030524 (PMC9478284; doi:10.1097/MD.0000000000030524)
Supplement: Supplementary file 2 [file medi-101-e30524-s002.pdf]

Supplemental Table 2. Treatment modality and clinical course of patients with IPF in ICU

	Total N=41	Survivors (N=21)	Non-survivors N=20	*P*-value
Treatments				
Antibiotics	41 (100)	21 (100)	20 (100)	
Steroids	28 (68.3)	15 (71.4)	13 (65.0)	0.744
Use of vasopressor	20 (48.8)	8 (38.1)	12 (60.0)	0.217
CRRT	6 (14.6)	1 (3.2)	5 (25.0)	0.093
ECMO	2 (4.9)	0	2 (10.0)	0.232
High flow nasal cannula	25 (61.0)	16 (76.2)	9 (45.0)	0.058
Ventilator support	33 (80.5)	16 (76.2)	17 (85.0)	0.697
Clinical course				
Presence of acute kidney injury	14 (34.1)	6 (28.6)	8 (40.0)	0.529
Pneumothroax	4 (9.8)	1 (4.8)	3 (15.0)	0.343
Length of ICU stay, days	8 (4-17)	10 (4-24)	7 (2-14)	0.201
In-hospital mortality	30/41 (73.2)	10/21 (47.6)	20/20 (100)	<0.001

Data are presented as n (%) or the median (interquartile range).

CRRT = continuous renal replacement therapy, ECMO = extracorporeal membrane oxygenation, IPF = idiopathic pulmonary fibrosis.
